# Broad and flexible stable isotope niches in invasive non-native *Rattus* spp. in anthropogenic and natural habitats of central eastern Madagascar

**DOI:** 10.1186/s12898-017-0125-0

**Published:** 2017-04-17

**Authors:** Melanie Dammhahn, Toky M. Randriamoria, Steven M. Goodman

**Affiliations:** 1 Animal Ecology, Institute for Biochemistry and Biology, Faculty of Natural Sciences, University of Potsdam, Maulbeerallee 1, 14469 Potsdam, Germany; 2 Association Vahatra, BP 3972, 101 Antananarivo, Madagascar; 3 Mention Zoologie et Biodiversité Animale, Université d’Antananarivo, BP 906, 101 Antananarivo, Madagascar; 4 Field Museum of Natural History, 1400 South Lake Shore Drive, Chicago, IL 60605 USA

**Keywords:** Bayesian standard ellipse, Coexistence, Habitat use, Humid forest, Invasion ecology, Invasive species, *Rattus rattus*, *Rattus norvegicus*, Rodents, Fur, Stable carbon isotope, Stable nitrogen isotope

## Abstract

**Background:**

Rodents of the genus *Rattus* are among the most pervasive and successful invasive species, causing major vicissitudes in native ecological communities. A broad and flexible generalist diet has been suggested as key to the invasion success of *Rattus* spp. Here, we use an indirect approach to better understand foraging niche width, plasticity, and overlap within and between introduced *Rattus* spp. in anthropogenic habitats and natural humid forests of Madagascar.

**Results:**

Based on stable carbon and nitrogen isotope values measured in hair samples of 589 individual rodents, we found that *Rattus rattus* had an extremely wide foraging niche, encompassing the isotopic space covered by a complete endemic forest-dwelling Malagasy small mammal community. Comparisons of Bayesian standard ellipses, as well as (multivariate) mixed-modeling analyses, revealed that the stable isotope niche of *R*. *rattus* tended to change seasonally and differed between natural forests and anthropogenic habitats, indicating plasticity in feeding niches. In co-occurrence, *R*. *rattus* and *Rattus norvegicus* partitioned feeding niches. Isotopic mismatch of signatures of individual *R*. *rattus* and the habitat in which they were captured, indicate frequent dispersal movements for this species between natural forest and anthropogenic habitats.

**Conclusions:**

Since *R*. *rattus* are known to transmit a number of zoonoses, potentially affecting communities of endemic small mammals, as well as humans, these movements presumably increase transmission potential. Our results suggest that due to their generalist diet and potential movement between natural forest and anthropogenic habitats, *Rattus* spp. might affect native forest-dependent Malagasy rodents as competitors, predators, and disease vectors. The combination of these effects helps explain the invasion success of *Rattus* spp. and the detrimental effects of this genus on the endemic Malagasy rodent fauna.

**Electronic supplementary material:**

The online version of this article (doi:10.1186/s12898-017-0125-0) contains supplementary material, which is available to authorized users.

## Background

Invasion of ecosystems by non-native species is a critical contemporary conservation threat because these species change ecological interactions, modify ecosystem functionality, and even cause extinctions of indigenous species [1]. Hence, the study of patterns, mechanisms, and consequences of the introduction of non-indigenous organisms is a major field of interest in ecology (e.g. [1, 2]). Important questions include whether invasive species are characterized by a specific set of traits (e.g. [3]) and, if so, whether certain traits make invasive species particularly devastating for indigenous ecological communities. In mammals, for example, species with large litter size, frequent breeding, and long reproductive lifespan are more likely to establish populations and spread after introduction [4]. The presence of invasive species can change selection pressures and, hence, the evolution of native species by direct genetic interaction such as hybridization or introgression [5] or by altering ecological interactions in communities, such as direct and apparent competition, predation, and parasitism [1]. In extreme cases, invasive species drive indigenous species to extinction (e.g. [6, 7]) or lead to the disassembly of communities [8]. Invasive species that are ecological generalists most likely have the strongest impact on native communities.

Murid rodents of the genus *Rattus* are a pervasive example of an invasive group of animals that is widespread and acts as the jack of all ecological trades. The ability of these animals to live successfully with humans has facilitated their transport [9], and today the black or ship rat (*Rattus rattus*), the Norway rat (*Rattus norvegicus*), and the Pacific rat (*Rattus exulans*) are established in many ecosystems worldwide and are among the most widespread and problematic invasive animals [10, 11]. The introduction of these rats to different island communities has severely impacted ground-nesting bird colonies in many areas of the world (reviewed in [12]). Moreover, being alternative prey for apex predators, *Rattus* spp. may also indirectly affect other prey species via apparent competition [13, 14]. For example, on the western Indian Ocean islands of Europa and Juan de Nova, introduced rats prey on ground-nesting birds but also constitute a food source for both native and introduced predators, which also prey on birds, leading to hyperpredation processes [15]. Furthermore, new parasites and pathogens carried by introduced *Rattus* spp. negatively impact native communities (reviewed in [16]). In Madagascar, dietary overlap [17], as well as external and craniodental morphological characteristics [18], suggest interference and exploitation competition for the same resources between rats and indigenous rodents. Hence, invasive rats may affect endemic species by the combined effects of predation, competition, and disease transmission. Indigenous organisms with independent evolutionary histories are therefore the most vulnerable to these altered ecological interactions [1, 19].

Separated from mainland Africa and India for approximately 90 million years [20], Madagascar has several independent terrestrial mammal radiations represented by four taxonomic orders: Afrosoricida, Carnivora, Primates, and Rodentia [21]. Two species of invasive rats, *R. rattus* and *R. norvegicus*, have been introduced to the island [22]. *Rattus rattus*, considered in the top 100 of the world’s worst invasive organisms [23], is more pervasive than *R. norvegicus*, particularly outside of urban areas, and has been able to colonize different forest habitats across the island; *R. norvegicus* tends to be more synanthropic [22]. Malagasy populations of *R. rattus* are from two successful introduction events from the same source population [24]. Seafarers from the Arabian Peninsula with *entrepôts* in eastern Africa and on Indian Ocean islands brought the species to Madagascar during the Middle Ages, when there was an increase in the movements of humans and trade goods across the Indian Ocean world [24, 25].

Today, *R. rattus* has overrun Madagascar [26] and occurs in virtually all natural and synanthropic habitats [27], representing >95% of rodent captures in certain natural forests, agricultural fields, and village settings [28, 29]. In areas where *R. rattus* have colonized natural habitats, which range from degraded to intact forests, there is an apparent concordant decline in endemic rodents of the subfamily Nesomyinae, which are strictly forest dwelling. However, direct impact of *R. rattus* on the endemic Malagasy mammalian fauna has been difficult to assess to date [18, 19], partly due to a lack of information on diet composition and habitat use of *R. rattus*. The main aim of this study was to investigate the trophic ecology of invasive *R. rattus* and *R. norvegicus* in natural humid forests, where they coexist with native mammals, and in anthropogenic village settings by using stable carbon and nitrogen isotope values of hair to assess indirectly trophic niche variation.

Stable isotope analyses of tissues, such as hair, have a considerable advantage over traditionally used methods of measuring diet such as fecal and stomach content analyses, because such samples provide information about diet integrated over several weeks (e.g. [30]). Metabolically inert hair reflects the diet of an individual for the period when the animal is replacing a large proportion of its fur [31, 32], which in laboratory *R*. *rattus* is ca. 40 days [33]. Other laboratory experiments with controlled diets revealed that in the fur of *R*. *norvegicus*, isotopic half-lives and retention times (the time taken to replace isotopes of different sources in tissues) for stable carbon and nitrogen ranges from 65 to 100 days [34]. If food sources available to a consumer differ in stable isotope signatures, the stable isotope niche (i.e. the area covered by individual consumer signatures in stable isotope bi-plots) can be used as a proxy for the trophic niche [35–37]. For example, animals feeding at several trophic levels such as omnivores have a larger range of stable nitrogen values; whereas variation in stable carbon values of the consumer mainly reflects variation in basal resources, such as different carbon cycles of plants. This approach is not without caveats because capturing all sources of variation in stable isotopes is difficult (e.g. [38, 39]). However, several empirical and experimental validations suggest that stable isotope niches can reflect both quantitative and qualitative aspects of trophic niches [37, 40]. For example, comparing stable isotope and stomach content analyses, Rodriguez and Herrera [41] showed that isotopic niches were an accurate measure of trophic niche width of *R. rattus* living on islands in the Gulf of California, USA.

In this study, we analyzed stable carbon and nitrogen isotope values in hair samples collected from individual *Rattus* spp. in different habitat types in central eastern Madagascar, which included agricultural fields, anthropogenic steppe, and natural humid forest formations (ranging from degraded to relatively intact). Specifically, we addressed the following hypotheses:

1. Invasive *R*. *rattus* has a generalist diet. Both in its native and invasive range, *R*. *rattus* is known to be an omnivorous generalist feeding on invertebrates, vertebrates, and plants (e.g. [42–49]), which has been shown by fecal and stomach content analyses and stable isotope analyses [41, 50, 51]. Therefore, we expect that *R*. *rattus* has a large stable isotope space ranging over several trophic levels and including various basal source pools.
2. Invasive *R*. *rattus* has a flexible diet. We predict that these omnivorous and generalist animals adjust their feeding niche to food availability and, therefore, occupy different stable isotope niches in natural and anthropogenic habitats. Since food sources are more diverse in forest habitats, we expect *R*. *rattus* to occupy wider stable isotope niches in natural forest settings as compared to anthropogenic steppe and agricultural fields. Moreover, we predict narrower stable isotope niches in the more food-rich wet season in comparison to the food-limited dry season [52].
3. Individual *R*. *rattus* disperse, traversing different habitat types. We expect a certain proportion of individuals to be isotopically mismatched to the habitat in which they were captured. Forest habitats, as the sampled evergreen mid-elevation humid forest, are dominated by C_3_ plants. Agricultural fields of a variety of crops (e.g., banana, sugar cane, maize, cassava, and rice) and anthropogenic steppe (secondary vegetation regrown after slash-and-burn agriculture) also have C_4_ plants (more details in Additional file 1: Text S1). C_3_ plants generally have lower δ^13^C values than C_4_ plants (global averages: −28 vs −14‰: [53, 54]). Therefore, we assume that natural forests differ from anthropogenic steppe and agricultural fields in δ^13^C baselines. Consequently, we predict *R*. *rattus* sampled in non-forested areas to have higher δ^13^C values than those from natural forests. If individuals move between habitat types, they should be isotopically mismatched particularly in δ^13^C. Since males are often cited as the dispersing sex [55], we expect a disproportional number of young adult males to be isotopically mismatched.
4. In sympatry, invasive *R*. *norvegicus* and *R*. *rattus* partition their feeding niches. Feeding niche partitioning is one of the principal mechanisms maintaining coexistence of congeneric species [56]. Previous studies revealed that in co-occurrence, morphologically smaller *Rattus* spp. have wider feeding niches than larger congeners [42]. Therefore, we predict that *R*. *rattus* (Madagascar adult animals, mean total body length 365.0 mm, mean body mass 116.2 g, [22]) has a wider stable isotope niche than the larger *R*. *norvegicus* (Madagascar adult animals, mean total body length 405.0 mm, mean body mass 259.0 g, [22]) [18].

## Methods

### Field sites and fieldwork

**Fig. 1:**
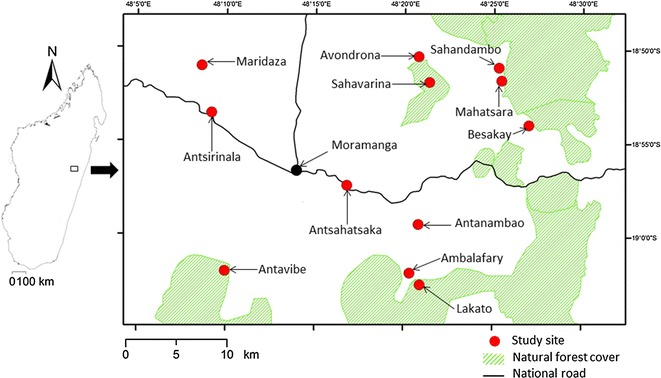
Sampling sites of *Rattus* spp. in the Moramanga District (Alaotra Mangoro Region) of central eastern Madagascar. The town of Moramanga is indicated by a *black dot*

Non-primate terrestrial small mammal surveys were carried out at 12 sites from July 2013 to March 2015 in the Moramanga District (Alaotra Mangoro Region) of central eastern Madagascar (Fig. 1; Additional file 1: Text S1, Additional file 2: Table S1). The work was conducted at three different types of sites:

1. Natural humid forest sites (n = 4): Antavibe, Avondrona, Lakato, and Sahandambo.
2. Villages sites (n = 5): Ambalafary, Antanambao, Antsahatsaka, Antsirinala, and Maridaza.
3. Sites with a combination of natural humid forest and villages (n = 3): Besakay, Mahatsara, and Sahavarina.

### Habitat classification

In accordance with the aims of this project, the habitats at the different study sites were classified as:

(1) Evergreen mid-elevation humid forest from 800 to 1800 m [43]. (2) Anthropogenic steppe or shrub thickets. This habitat included a formation known as *savoka*, referring to secondary vegetation, generally of non-native and invasive plants, that grow after removal of natural forest and at least at the initial stages associated with slash-and-burn agriculture (*tavy*). At such sites, within a few seasons of planting, the soils are depleted, rendering such areas poor for agriculture production. In turn, these sites are often abandoned, allowing the colonization of often dense secondary vegetation. (3) Village settings, which include habitats around houses, agricultural fields, grain storage buildings, and associated immediate areas. The agricultural zones include those with hill rice, paddy rice, and a variety of other crops (e.g. banana, sugar cane, maize, cassava, and rice).

### Trapping and sampling

Based on satellite images, each site was delimited into an area of 700 × 700 m or 49 ha. The number of trap lines installed in each habitat type per site was proportional to the representation of the habitat in the 49 ha parcel. Trap lines were installed a minimum distance of 60 m from each other.

Three different trap types were employed: BTS traps, 30 × 10 × 10 cm (BTS Company, Besançon, France); National traps, 39.2 × 12.3 × 12.3 cm (Tomahawk Trap Company, Hazelhurst, Wisconsin, USA); and Sherman traps, 22.5 × 8.6 × 7.4 cm (H.B. Sherman Traps Inc., Tallahassee, Florida, USA). Within each 49 ha site, 120 traps were distributed into 10 trap lines, each composed of 12 traps alternated as BTS-National-Sherman and the series repeated four times. Adjacent traps within a single line were separated from one another by a distance of 8–10 m. Traps were baited daily with a mixture of peanut butter and fresh cassava, visited in the morning and in the late afternoon, and during the latter period fresh bait was added and the previous bait removed.

A few milligrams of hair were collected from the back of each captured *Rattus* spp. (for sample sizes see Table 1). Each sample was placed in an Eppendorf tube, bearing the unique specimen number of the individual, and then exposed to indirect sunlight for about 15 min for thorough drying. To measure the isotopic variability in plants and soils of the habitat, i.e. an isotope habitat baseline, plant (n = 57) and soil (n = 49) samples were collected in close vicinity to the trap lines within each 49 ha site. At each study site, soil samples without leaf litter were collected in a defined position at 27 cm depth; plant samples were obtained in a more random manner and included leaves of the dominant floristic species, including C_3_ and C_4_ plants, during the season of animal capture at a particular site.

**Table 1:** Summary of trapping results and sampling for stable isotope analyses of *R. rattus and R*. *norvegicus* in the three habitat types and by site

Type of site	Site name	Season	*R*. *rattus*			*R*. *norvegicus*		
			Forest	Anthropogenic steppe	Agricultural field	Forest	Anthropogenic steppe	Agricultural field
Natural forest	Antavibe	Dry	60 (26)	–	–	–	–	–
		Wet	15 (11)	–	–	–	–	–
	Avondrona	Dry	11 (11)	–	–	–	–	–
		Wet	70 (40)	–	–	–	–	–
	Lakato	Dry	130 (1)	–	–	1 (1)	–	–
		Wet	28 (26)	–	–	–	–	–
	Sahandambo	Wet	47 (15)	–	–	–	–	–
Village	Ambalafary	Dry	–	156 (37)	16 (4)	–	2 (2)	–
		Wet	–	66 (64)	7 (6)	–	–	–
	Antanambao	Wet	–	62 (29)	21 (4)	–	–	1 (1)
	Antsahatsaka	Dry	–	68 (3)	95 (1)	–	–	7 (7)
		Wet	–	30 (13)	22 (8)	–	–	3 (3)
	Antsirinala	Dry	–	22 (12)	15 (10)	–	–	1 (1)
	Maridaza	Wet	–	82 (40)	8 (7)	–	2 (2)	4 (4)
Natural forest-village	Besakay	Dry	90 (0)	104 (2)	35 (0)	–	–	–
		Wet	8 (1)	60 (16)	21 (7)	–	1 (1)	–
		Dry	34 (24)	94 (38)	28 (13)	–	–	–
	Mahatsara	Wet	3 (3)	33 (3)	31 (3)	–	–	–
	Sahavarina	Dry	17 (10)	20 (11)	60 (43)	–	–	2 (2)
		Wet	8 (8)	2 (2)	17 (13)	–	–	–

As not all captured animals were sampled for stable isotope analyses, these different values are presented. The first figure under the habitat types is the number of captured individuals based on 600 standard live trap-nights per site and the second figure, in parentheses, is the number of individuals included in the stable isotope analyses. In the “natural forest” sites, traps were limited to forest habitat; in “village” sites, traps were in anthropogenic steppe and agricultural fields; and in “natural forest-village” sites, traps were in all three habitats. -: not present

### Ethics statement

The study has been conducted in accordance with the Institut Pasteur (Paris) guidelines (http://www.pasteur.fr/ip/easysite/pasteur/en/institut-pasteur/ethics-charter) for animal husbandry and experiments. As no national committee for animal welfare existed on Madagascar during the period of this study, the protocol was approved and validated by an Ad hoc committee of the Institut Pasteur de Madagascar, which included representatives from the Veterinary School of the University of Antananarivo and the Madagascar Ministry of Livestock.

### Stable isotope analyses

Samples were analyzed at the Centre for Stable Isotopes Research and Analyses (KOSI) in Göttingen, Germany. Prior to analyses, each hair sample was cleaned with ethanol and all samples were oven dried at 60 °C until weight was constant. Lipid extraction was not performed for hair samples because C:N ratio was 3.3 ± 0.17 (mean ± SD) which was below that suggested for extraction (3.5; [57]). Plant samples were ground and homogenized with a ball mill. Soil samples were homogenized. For determination of δ^13^C and δ^15^N, approximately 1 mg of total hair, homogenized soil, and plants was enclosed into tin capsules, and subsequently processed through an isotope ratio mass spectrometer (Delta Plus, Finnigan MAT, Bremen, Germany) in an online-system after passage through an element analyzer (NA 1110, Carlo Erba, Milan, Italy). The isotope data are presented in ‰ as δ^15^N relative to nitrogen in air or δ^13^C relative to Pee Dee Belemnite calculated as follows:

$$ \delta {\text{X}}\left( \permil \right) = \left[ \left( {\text{R}}_{\text{sample}} / {\text{R}}_{\text{standard}}  \right) - 1 \right] \times 1000 $$

where δX is δ^15^N or δ^13^C, and R is the respective ^15^N/^14^N or ^13^C/^12^C ratio. Analytical precision (±SD) was calculated based on 300 acetanilide laboratory working standard (KOSI2, Göttingen, Germany) replicates (two after each 10 unknowns) and was 0.12‰ for δ^15^N and 0.12‰ for δ^13^C.

### Statistical analyses

To compare stable isotope niches among *R. rattus* sampled in different habitats, we used two approaches. A description of our habitat classification is presented above. First, based on the R package *SIAR* [58, 59], we calculated standard ellipses and convex hulls for *R. rattus* within each habitat and tested for differences in size of stable isotope niches among habitats. Thereafter, we calculated overlap between standard ellipses and convex hulls among *R. rattus* sampled in different habitats. Using Bayesian estimates of standard ellipses (based on 10,000 posterior draws and default settings of *SIAR*), we compared the size of stable isotope niche widths among *R. rattus* of different habitats. This technique has two advantages: (1) it is relatively robust against unequal sample sizes and (2) it includes measures of uncertainty around the estimates [59]. We followed the same procedure to estimate size and overlap of stable isotope niches between *R. rattus* and *R. norvegicus*; these comparisons were based only on samples obtained from locations where the two species co-occurred (Table 1).

Second, to test for differences in stable isotope niches among *R*. *rattus* sampled in different habitats, we used multivariate mixed models with Gaussian error distributions run with the R package *MCMCglmm* (Markov-chain Monte-Carlo generalized linear mixed models, [60]). These models use a Bayesian approach and provide a control for heterogeneity in variances and sample sizes between sampling sites by adding the random effect sampling site (specified as a random intercept) and test for differences between habitats specified as fixed effects. In addition, we entered season (defined as dry and wet, see below), as well as sex and age classes (defined as non-reproductive and reproductive, see below) to test whether these factors explain variation in stable isotope values of *R. rattus*. Aspects of season were derived from climatological data of the general study area and two periods were recognized: (1) dry season from July to October and (2) wet season from December to April. We classified individuals into two age classes based on breeding status: (1) non-reproductive—males with small abdominal testes and undeveloped epididymides and females with imperforated vagina and small mammae, and (2) reproductive—males with scrotal testes and developed epididymides and females with embryos, large mammae, and/or lactating. Since males are documented to be the dispersing sex in *R. rattus* [55], and, in general, rodents tend to disperse before breeding [61], we further entered an interaction between sex and age class, specified as a fixed effect, in this multivariate mixed model.

We set slightly informative priors by dividing the total phenotypic variance of δ^13^C values and δ^15^N values by the number of random effects in the model and set a low degree of belief (nu = 1) [60]. We used 200,500 iterations, a thinning interval of 200, and a burn-in of 500, which resulted in low temporal autocorrelation between estimates of subsequent models. Subsequently, we used univariate linear mixed models and the procedures described in Zuur et al. [62] to test for differences in δ^13^C and δ^15^N among *R. rattus* sampled in different habitats. We obtained the explained variances (pseudo-R^2^) using the approach suggested by Nakagawa and Schielzeth [63]. We followed the same method to test for differences between *R. rattus* and *R. norvegicus* stable isotope niches, but we included only habitat type as a fixed effect.

To explore potential stable isotope mismatch between individual signatures and the habitat a given animal was trapped, we first tested our assumption of habitat-specific stable isotope baselines. Using soil and plant samples collected along trapping transects (see above), we tested whether habitat types, specified as fixed effects, differ in δ^13^C and δ^15^N, respectively, using restricted maximum likelihood linear mixed modeling (LMM) with normal errors and the R-package *lme4* [62]. The sampling site was entered as random effect (specified as random intercept) in this model. Assuming that (1) habitats differ in stable isotope baselines and (2) *R*. *rattus* sampled in different habitats differ isotopically, we first classified all individuals outside of the statistical non-outlier range of the population of a specific habitat as stable isotopically mismatched. Here, statistical outliers were δ^13^C values (x) with either x < Q1 − 1.5 * (Q3 − Q1) or x > Q3 + 1.5 * (Q3 − Q1), with Q1 = first quartile and Q3 = third quartile of the distribution of individual δ^13^C distributions. Forested habitats are dominated by C_3_ plants, while agricultural fields and anthropogenic steppe have C_4_ plants. Therefore, we based this classification only on the stable carbon isotope.

Further, we used linear discriminant function analysis with leave one out cross (jackknifed) validation, run with the *lda* function of the R-package *MASS*, to (1) quantify overall and habitat-specific percentages of correct classifications of individual *R*. *rattus* to their habitat of origin, and (2) to identify misclassified individuals. Since sampling sites were isotopically heterogeneous, we corrected individual *R*. *rattus* isotope signatures by the isotope baseline of each sampling site (δ^15^N_cor_ = δ^15^N_rat_ − δ^15^N_plants_ and δ^13^C_cor_ = δ^13^C_rat_ − δ^13^C_plants_), with δ^15^N_plants_ and δ^13^C_plants_ being the median of all plant samples collected in forested areas at each site. We used only one habitat type for this baseline correction to retain habitat-type specific variation and selected forest because we were mainly interested in detecting signs of movement between forested and human-modified habitats.

Finally, using generalized mixed effect models (GLMM) with binomial error distribution and sampling site as a random effect (specified as random intercept), we tested whether the probability of being a statistical outlier for δ^13^C values and of being misclassified, respectively, was higher for males than for females and higher for non-reproductive than for reproductive individuals. Since males are assumed to be the dispersing sex, particularly from natal areas, and such movements occur before breeding (see above), we further entered an interaction between sex and age class specified as a fixed effect in these models. Moreover, we entered habitat type as a fixed effect to control for potential habitat type differences. For each model, we checked whether residuals of the model followed a Gaussian distribution. All data were analyzed using R 2.15 (The R Foundation for Statistical Computing, Vienna, Austria, http://www.r-project.org) and the specified packages. Values of *P* were two tailed throughout and the accepted significance level was *P* < 0.05.

## Results

### Habitats and sites in which *Rattus* spp. were captured

In total, 565 individuals of *R. rattus* and 24 individuals of *R. norvegicus* were sampled across the different sites and habitat types [Table 1, raw data at Dryad Digital Repository: http://dx.doi.org/10.5061/dryad.j04ff)]. *Rattus rattus* was present in all habitats, whereas *R*. *norvegicus* was mainly found in agricultural fields and less frequently in anthropogenic steppe. Lakato was the only forested site in which *R*. *norvegicus* was captured.

### Within-species niche variation in *R. rattus*

**Fig. 2:**
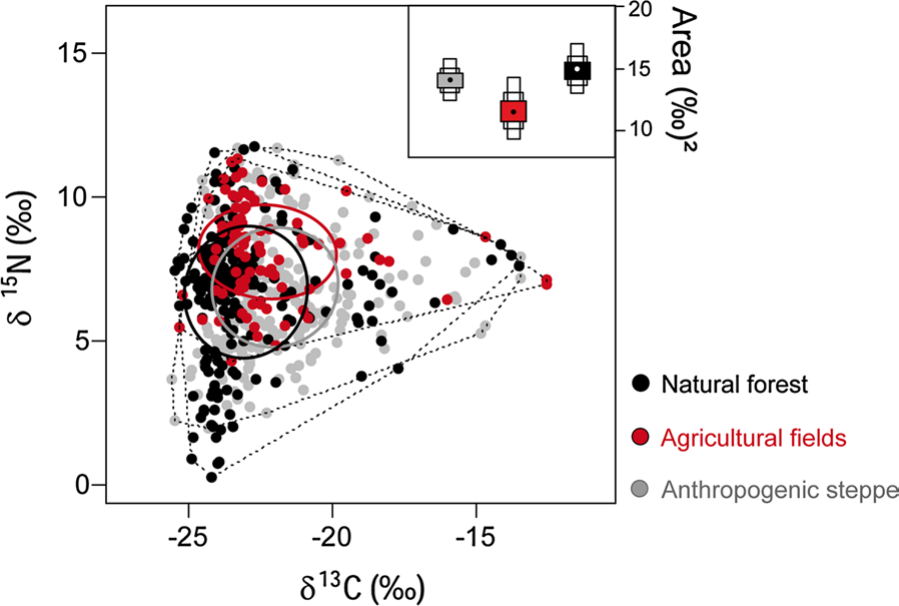
Scatterplot—stable carbon and nitrogen values of individual *R. rattus* from agricultural fields, natural forests, and anthropogenic steppe. Polygons indicate convex hull areas (*broken lines*); *ellipses* indicate standard ellipse areas (*solid lines*). Boxplot—stable isotope niche areas of *R*. *rattus* from natural forests and anthropogenic steppe were wider than that of *R*. *rattus* from agricultural fields, based on Bayesian standard ellipse estimates with *SIAR*. Shown are posterior modes (*dot*) and the 25, 75, and 95% credibility intervals of posterior distributions of 10,000 simulations (*boxes*)

The stable isotope niche width of *R. rattus* was large in all sampled habitat types (Fig. 2; Additional file 3: Table S2). This species had a wider stable isotope niche in natural forests as compared to anthropogenic steppe (comparison of standard ellipses: *P* = 0.032) and tended to cover a wider niche in natural forests as compared to agricultural fields (*P* = 0.072) (Fig. 2; Additional file 3: Table S2). The stable isotope niches of *R*. *rattus* in agricultural fields, natural forests, and anthropogenic steppe showed notable overlap (45–72%; Fig. 2; Additional file 4: Table S3).

Stable isotope niches in *R. rattus* differed between natural forests and agricultural fields (multivariate mixed model: *P* = 0.038, Table 2) but not between natural forests and anthropogenic steppe (*P* = 0.14). *Rattus rattus* sampled in the various habitats differed in overall δ^13^C (Table 3, *pseudo*-*R* ^2^ = 0.12): those in agricultural fields tended to have higher δ^13^C than those sampled in natural forests, and those in anthropogenic steppe had higher δ^13^C than those sampled in natural forests (Table 3). The various individuals of this species showed no differences between habitats in δ^15^N (Table 3).

**Table 2:** Results of multivariate Bayesian mixed models on within-species niche variation in *R. rattus*

Parameter	Posterior mean	l-95% CI	u-95% CI	*P*
Intercept δ^13^C	*−22.44*	*−23.17*	*−21.71*	*<0.001*
Intercept δ^15^N	*7.20*	*5.97*	*8.40*	*<0.001*
Anthropogenic steppe^a^	0.24	−0.05	0.59	0.138
Agricultural field^a^	*0.40*	*0.03*	*0.75*	*0.038*

Shown are posterior means, lower, and upper 95% credibility intervals and *P* values, which are based on 10,000 simulations

Significant results are marked in italic

Reference level is ^a^natural forest

**Table 3:** Results of univariate LMMs for δ^13^C and δ^15^N on within-species niche variation in *R. rattus*

Parameter	β ± SE	*t*	*P*	*Χ* ^2^*	P*
δ^13^C					
Intercept	*−22.88* *±* *0.30*	*75.96*	*<0.001*		
Anthropogenic steppe^a^	*0.71* *±* *0.26*	*2.75*	*0.006*		
Agricultural field^a^	0.58 ± 0.32	1.80	0.072	*7.60*	*0.022*
δ^15^N					
Intercept	*7.02* *±* *0.54*	*13.11*	*<0.001*		
Anthropogenic steppe^a^	0.07 ± 0.18	0.39	0.696		
Agricultural field^a^	0.34 ± 0.22	1.58	0.114	2.80	0.246

Significant results are marked in italic

Reference level is ^a^natural forest

*Χ ^*2*^ and *P* values are based on log-likelihood-ratio tests (LRT) comparing models with and without the main effect of habitat type with *df* = 2

When controlling for habitat in *R*. *rattus*, no difference was found in stable isotope niches between males and females (*P* = 0.17), and between non-reproductive and reproductive individuals (*P* = 0.92) (Additional file 5: Table S4). The interaction between sex and age class did not improve model fit (ΔDIC < 2) and was removed from the model. Stable isotope niches of *R*. *rattus* tended to be smaller in the wet season as compared to the dry season (posterior mean = −0.23; CI: −0.49, 0.04, *P* = 0.088) (Additional file 5: Table S4). Univariate models for δ^13^C and δ^15^N revealed no effect of sex, age class, and season for both stable isotopes (Additional file 6: Table S5). Moreover, including the interaction between sex and age class did not improve model fit. *Rattus rattus* sampled in different habitats differed only in δ^13^C values, more specifically, those sampled in anthropogenic steppe and agricultural fields had higher δ^13^C values than those sampled in natural forests (Additional file 6: Table S5).

### Habitat mismatching

First, we tested our assumption of isotopic difference between habitat types. Controlling for sampling site, we found that *R*. *rattus* sampled in natural forest had lower δ^13^C values than those from anthropogenic steppe and agricultural fields (see above). Taking only trapping sites into account where all three habitat types were represented and with sufficient sample sizes (Ambalafary, Besakay, and Sahavarina), *R*. *rattus* from natural forest had lower δ^13^C values than those sampled in anthropogenic steppe and agricultural fields (Additional file 7: Table S6). Moreover, habitat baseline measurements differed between habitat types (soil: *Χ* ^2^ = 6.37, *df* = 2, *P* < 0.041; plants: *Χ* ^2^ = 15.64, *df* = 2, *P* < 0.001, Additional file 8: Table S7, Additional file 9: Table S8). Soil samples tended to have lower δ^13^C values in natural forest as compared to anthropogenic steppe (*β* = −1.18 ± 0.63, *t* = 1.85, *P* = 0.064) and were lower in δ^13^C values in forests than in agricultural fields (*β* = −1.41 ± 0.62, *t* = 2.29, *P* = 0.022). In addition, forest plants had lower δ^13^C values than plants collected in or near agricultural fields (*β* = −6.95 ± 1.70, *t* = 4.09, *P* < 0.001), but did not differ from plants collected in anthropogenic steppe (*β* = −1.53 ± 1.01, *t* = 1.52, *P* = 0.13).

Based on stable isotope values, we found indirect evidence of dispersal movements of *R*. *rattus* between habitat types. This species varied widely in δ^13^C values with many individuals having values outside the non-outlier range of the habitat in which they were trapped (Fig. 2). These were not predominantly young males (interaction sex × age class: *Χ* ^2^ = 1.67, *df* = 1, *P* = 0.20), but included females, as well as non-reproductive and reproductive animals.

Stable isotope signatures of individual *R*. *rattus* allowed moderate prediction inference of the habitat type in which the animal was captured. Discriminant function 1 explained 90.4% of the total variance and habitat type was correctly classified in 54.6% of the cases. Classification was similar for *R*. *rattus* sampled in natural forest (59%) and anthropogenic steppe (67%), but individuals sampled from agricultural fields could not be classified (4% correctly classified). The majority of misclassified anthropogenic steppe samples were classified as from natural forest or vice versa. Misclassification of natural forest and anthropogenic steppe samples to agricultural fields was rare (natural forest: 1/207; anthropogenic steppe: 11/246). Using only samples from natural forest and agricultural fields, correct classification of forest samples was high (90%), but those from agricultural fields remained moderate (41%). Neither sex (*Χ* ^2^ = 0.61, *df* = 1, *P* = 0.44), age class (*Χ* ^2^ = 0.70, *df* = 1, *P* = 0.40) or the interaction between sex and age class (*Χ* ^2^ = 1.29, *df* = 1, *P* = 0.26) affected the probability of an individual being misclassified in the DFA.

### Niche differentiation between *R. rattus* and *R*. *norvegicus*

**Fig. 3:**
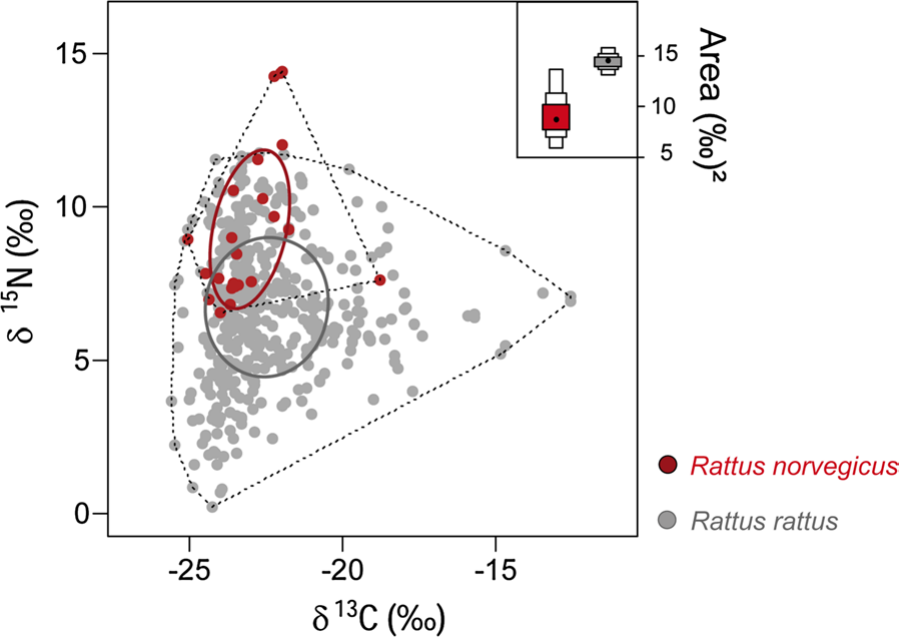
Scatterplot—stable carbon and nitrogen values of individual co-occurring *R. rattus* and *R*. *norvegicus*. Polygons indicate convex hull areas (*broken lines*) and *ellipses* indicate standard ellipse areas (*solid lines*). Boxplot—stable isotope niches of *R*. *rattus* and *R*. *norvegicus* differ markedly in width, based on Bayesian standard ellipse estimates with *SIAR*. Shown are posterior modes (*dot*) and the 25, 75, and 95% credibility intervals of posterior distributions of 10,000 simulations (*boxes*)

In co-occurrence, stable isotope niches differed between *Rattus* spp. (multivariate mixed model: *P* < 0.001) but not between habitats (Table 4; Fig. 3). This difference is due to variation between species in δ^15^N values (*Χ* ^2^ = 21.16, *df* = 1, *P* < 0.001, *pseudo*-*R* ^2^ = 0.58), but not in δ^13^C values (*Χ* ^2^ = 0.71, *df* = 1, *P* = 0.40, *pseudo*-*R* ^2^ = 0.08) (Table 5). Combining all habitat types, in which the two species were captured in sympatry, *R*. *rattus* covered a larger stable isotope niche width than *R*. *norvegicus* (Fig. 3); the areas of standard ellipses and convex hulls were 14.3 and 90.4‰^2^ for *R*. *rattus* and 9.4 and 26.7‰^2^ for *R*. *norvegicus*. The core stable isotope niche (estimated as a standard ellipse) of *R*. *rattus* was larger than that of *R*. *norvegicus* combining all habitat types (*P* = 0.027). *Rattus rattus* overlapped with 35% of the core stable isotope niche of *R*. *norvegicus*, whereas only 24% of core isotope niche of *R*. *rattus* was shared with *R*. *norvegicus*.

**Table 4:** Results of multivariate Bayesian mixed models on niche differentiation between *R. rattus* and *R*. *norvegicus*

Parameter	Posterior mean	l-95% CI	u-95% CI	*P*
Intercept δ^13^C	*−21.70*	*−22.68*	*−20.72*	*<0.001*
Intercept δ^15^N	*7.99*	*6.77*	*9.27*	*<0.001*
Species^a^	*−1.03*	*−1.56*	*−0.45*	*<0.001*
Anthropogenic steppe^b^	0.25	−0.06	0.55	0.116
Agricultural field^b^	0.29	−0.05	0.66	0.126

Shown are posterior means, lower and upper 95% credibility intervals, and *P* values, which are based on 10,000 simulations

Significant results are marked in italic

Reference levels are ^a^ *R*. *norvegicus* and ^b^ natural forest

**Table 5:** Results of univariate LMMs for δ^13^C and δ^15^N on niche differentiation between *R. rattus* and *R*. *norvegicus*

Parameter	β ± SE	*t*	*P*	*Χ* ^2^*	P*
δ^13^C					
Intercept	*−23.42* *±* *0.49*	*47.70*	*<0.001*		
Species^a^	0.34 ± 0.43	0.78	0.433	0.71	0.39
Anthropogenic steppe^b^	*0.62* *±* *0.24*	*2.64*	*0.008*		
Agricultural field^b^	*0.71* *±* *0.29*	*2.43*	*0.015*	*7.92* ^*c*^	*0.019* ^*c*^
δ^15^N					
Intercept	*8.58* *±* *0.83*	*10.25*	*<0.001*		
Species^a^	*−1.60* *±* *0.36*	*4.45*	*<0.001*	*21.16*	*<0.001*
Anthropogenic steppe^b^	0.05 ± 0.20	0.26	0.796		
Agricultural field^b^	0.13 ± 0.24	0.54	0.585	0.31	0.85

Significant results are marked in italic

Reference levels are ^a^ *R*. *norvegicus* and ^b^ natural forest

*Χ ^*2*^ and *P* values are based on log-likelihood-ratio tests (LRT) comparing models with and without the respective term with *df* = 1; ^c^ for the main effect of habitat type with *df* = 2

## Discussion

Species in the genus *Rattus* are among the most pervasive and successful invasive mammals in the world and responsible for major changes in native ecological communities in areas where they have been introduced. Besides dispersal and life-history characteristics of these animals [4], a flexible generalist diet appears to be another important aspect that explains their successful invasion of non-native ecosystems [41]. Here, we used stable isotope analyses to study feeding niches of introduced *Rattus* on Madagascar and found that *R*. *rattus* had an extremely broad feeding niche (see next section). In co-occurrence, *R*. *rattus* and *R*. *norvegicus* appear to partition their feeding niches, enlarging the trophic niche space covered by the genus. Moreover, we found indirect evidence that individual *R*. *rattus* disperse between anthropogenic habitats and natural forest. These results suggest that due to their generalist diet, rats presumably affect endemic small mammal species as competitors and predators, and, as shown by other studies, as vectors of parasites and zoonotic diseases [64, 65]. The combination of these effects might explain both the invasion success of rats, as well as their detrimental effects on the native fauna. Below we discuss our results in more detail.

### Introduced *R. rattus* have an exceedingly wide feeding niche

Feeding niches of *R. rattus* on Madagascar were notably wide. As indicated by variation in δ^15^N values, the diet of this species might include food sources from several trophic levels, with trophic levels being generally separated by 3–5‰ in δ^15^N [66–68]. Variation in δ^13^C values further suggests that *R*. *rattus* incorporated C_3_- and C_4_/CAM-based source pools in their diets. Forest ecosystems are dominated by C_3_ plants, which have lower δ^13^C values than C_4_ plants (global average −28 vs −14‰; [54]; −29.8 ± 1.6‰ (mean ± SD) in our study) and drought-adapted CAM plants [53]. Overall, the feeding niche of this introduced rodent was notably larger than that of any native Malagasy mammal species or genus studied so far (e.g. [69–75]), and about three-times larger than the complete native small mammal community of Nesomyinae rodents and Tenrecidae tenrecs at a site in the Malagasy Central Highlands [71].

Many other dietary studies of *R. rattus* outside of Madagascar, both in its native and invasive range, described the species as an omnivorous generalist at the population level. For example, it is known to feed on earthworms [43–45], terrestrial mollusks [46], crabs [47], snails [49], arthropods [42], and birds [49]. In the Hawaiian Islands, *R*. *rattus* contributes to the dispersal of some native and non-native seeds, including the highly invasive shrub *Clidemia hirta* [42]. Moreover, stable isotope analyses have shown that invasive *Rattus* spp. are characterized by a generalist diet (*R. rattus*: [41, 50]; *R. norvegicus*: [51]).

Our stable isotope samples were derived from specimens originating from different habitat types, including natural forest and various forms of anthropogenic steppe, ranging from grasslands to shrublands, as well as agricultural fields with cassava, rice, legumes, maize, sugar cane, and banana. Nevertheless, the trophic space covered by *R*. *rattus* sampled in these different habitats overlapped largely. Only animals from agricultural fields had a narrower feeding niche than those from natural forest or anthropogenic steppe. These differences were driven by variation in δ^13^C values, but not in δ^15^N values, reflecting the C_3_ and C_4_ plant dominated source pools (see above). During the wet season, when food availability is presumed to be higher [52], *R*. *rattus* tended to have narrower feeding niches as compared to the more food-limited dry season. Hence, a generalist diet with high among-individual variation appears to be a common and habitat-independent feature of *R*. *rattus* trophic ecology in the habitats we studied.

*Rattus rattus* is similar in external and craniodental morphological traits to some endemic forest-dwelling rodents of the subfamily Nesomyinae, namely terrestrial *Nesomys* and *Gymnuromys* and scansorial *Eliurus* spp. [18], and they might compete with these species for resources. Indeed, inference of diet overlap has been found based on seeds excavated from burrows of nesomyines and *R. rattus* in natural forest of the Parc National d’Andringitra, in central southern Madagascar [17]. Future studies should focus more closely on food resource and trophic niche overlap between *R. rattus* and nesomyines to assess interference and exploitation competition between these animals. In summary, an extraordinary large trophic niche space of *R*. *rattus* might explain its invasion success of different habitats and ecosystems, as this allows (1) establishing new populations under novel environmental conditions and (2) population maintenance due to reduced intra-specific competition.

### Introduced *Rattus* spp. partition generalist feeding niches

Feeding niche partitioning is one of the principal mechanisms maintaining stable coexistence of species in communities. Particularly, the coexistence of closely related congeneric taxa has attracted much attention from ecologists because these species should show closer similarities in their feeding niches than more distantly related species [76]. However, studying coexistence mechanisms in extant communities is not always tractable and is shadowed by the “ghost of competition past” [77]. In the case of Madagascar, the presence of two species of introduced congeneric rodents, *R*. *norvegicus* and *R*. *rattus*, offers an excellent opportunity to study coexistence mechanisms at work, at least on a short-time scale. Here, we indirectly analyzed trophic niches of these two widespread species and found that they differ in feeding niche width. The larger and presumably more competitive *R*. *norvegicus* covers a smaller feeding niche than the smaller and presumably less competitive *R*. *rattus*. These results should be interpreted with caution because sample sizes for *R. norvegicus* was only moderate and not evenly spread over the habitat types. Nevertheless, we think that the results are not simply an artifact of differences in sample size between species, as the employed Bayesian approach and the standard ellipse comparisons are not particularly sensitive to heterogeneous sample sizes. Moreover, the stable isotope niches of both species only overlap moderately. Similarly, in the Hawaiian Islands, as revealed by stomach content analyses, the smaller *R*. *exulans* had a wider feeding niche than its larger congener *R*. *rattus* [42]. In the context of our Madagascar study, higher δ^15^N values in *R*. *norvegicus* might indicate a greater percentage of animal source food in their diet as compared to *R*. *rattus*. Besides this feeding niche partitioning, both species appear to segregate spatially on Madagascar, with *R. norvegicus* being mainly around villages and in anthropogenic habitats, rarely captured in natural forest, in contrast to *R*. *rattus* [22]. Stable isotope niche differentiation between invasive *Rattus* spp. enlarges the trophic space covered by the genus and, in turn, might increase the ecological effects of these species on native mammal communities.

### Stable isotope mismatch suggests movement between habitats

Within *R. rattus*, several non-reproductive and reproductive individuals of both sexes and at all sampling sites showed mismatching between their individual stable isotopic signature and that of the habitat where they were trapped. Differential resources use exploitation of C_3_ and C_4_ plants by individuals might theoretically lead to such a pattern for anthropogenic steppe and agricultural fields, where both types of plants exist, but cannot explain why certain individuals have higher δ^13^C values in natural forests, where only C_3_ plants occur. Therefore, we suggest that given general differences between habitat types in stable isotope baselines, as well as habitat-specific signatures of individuals of this species, these mismatches indicate that regardless of age and sex, animals move between habitats on a spatial scale of hundreds of meters to a few kilometers. In studies of *R*. *rattus* outside of Madagascar, males, particularly subadults, have been cited as the predominantly dispersing sex [55], and dispersal occurred mainly before the reproductive season [61]. Therefore, we anticipated males in particular to have a greater proportion of stable isotopic signatures outside the habitat-specific range in which they were captured. However, our results suggest that these mismatched signals occur in the different age and sex classes of *R*. *rattus* and with no strong seasonal component.

Hitherto, no study has tracked the exact movements of individual *R*. *rattus* on Madagascar. In geographical areas outside of Madagascar, where this species has been introduced, its home range size shows considerable variation between habitats. For example, Whisson et al. [78] found average home range sizes of 0.45 ha for females and 0.78 ha for males based on radio-telemetry over 2 months in a riparian habitat of California, which was similar to the findings of Hooker and Innes [79] of 0.49 ha for females and 1.1 ha for males in New Zealand. However, in a short-term 4-day radio-telemetry study in a New Zealand forest, males ranged up to 11 ha [80]. During one night, a given individual can travel up to 900 m [79] and traverse areas without vegetational cover approaching 500 m [81].

On Madagascar, movements of *R*. *rattus* between different types of anthropogenic habitats have been shown. For example, based on trapping studies at two east coast sites, this species shifted seasonally to villages, particularly around houses, during the period of rice harvest or food scarcity in secondary forested habitat [82]. Using Rhodamine B as a marker, Rahelinirina et al. [65] demonstrated that in eastern Madagascar individuals of this species move regularly between houses, adjacent sisal plantations, and irrigated rice fields, and up to 350 m over a 3-month period. Moreover, *R*. *rattus* is well known to occupy rice fields, to ravage crops [83], and then displace to higher ground when food resources decrease or burrows are flooded during the wet season [84]. Our indirect results confirm these observations, but we could not detect seasonal patterns in dispersal, presumably associated with our stable isotope measurements of hair integrating information over at least a period of several weeks.

We present evidence for *R*. *rattus* ranging between anthropogenic and natural forests; this aspect has several important implications. First, tracking resource availability by switching seasonally between habitats might provide rats another competitive advantage as compared to the endemic nesomyine rodents, which are largely forest-dependent. Second, *Rattus* spp. and their associated parasites are known to transmit a number of zoonotic diseases, which might negatively affect native communities and increase the invasion success of *Rattus* spp. (see [16] for a summary of parasite-related effects and consequences of invasion by *Rattus*). Such mobility increases the sphere of transmission potential of *Rattus*. For example, the fleas of *R*. *rattus* in some areas of Madagascar are a bubonic plague reservoir [85], and at a forested site in the Central Highlands, *Yersinia pestis* (plague bacteria) was isolated from endemic mammals, as well as *R*. *rattus* [27]. Investigations on the occurrence and morphology of trypomastigotes, a flea-transmitted parasite, suggest that introduced *R*. *rattus* might contribute to the decline of native endemic rodents by the transmission of this parasite [86]. In order to understand in better detail the role and associated probability of *R. rattus* to transmit endo- and ectoparasites between anthropogenic and natural habitats on the island, future studies should focus on tracking the movements of individual animals. Moreover, phylogeographical studies of parasites at a local scale [87] or capture-mark-recapture of ectoparasites [88] might provide further insight into the potential of *R*. *rattus* to introduce parasites and diseases into native mammalian communities.

## Conclusions

Although the analysis of stable isotopes allows only indirect inferences, our results suggest that on Madagascar introduced *Rattus* spp. might affect endemic forest-dependent rodents as competitors, predators, and disease vectors. *Rattus rattus* had an extremely broad and flexible feeding niche. Moreover, in co-occurrence *R*. *rattus* and *R*. *norvegicus* appear to partition their feeding niches, enlarging the trophic niche space covered by the genus. Movements between anthropogenic habitats and natural forests might increase the transmission potential of *R*. *rattus,* a well known vector of a number of zoonoses. The combination of these effects helps explain the invasion success of *Rattus* spp. and the detrimental effects of members of this genus on the endemic Malagasy rodent fauna.

## Additional files

**Additional file 1: Text S1.** Characterization of sampling sites.

**Additional file 2: Table S1.** Summary of the sampling period and geographic position for each sampling site.

**Additional file 3: Table S2.** Areas of standard ellipses and convex hulls for *Rattus rattus* in different habitats.

**Additional file 4: Table S3.** Pairwise overlap between stable isotope niches of *Rattus rattus* in different habitats.

**Additional file 5: Table S4.** Results of extended multivariate Bayesian mixed model for δ^13^C and δ^15^N of *Rattus rattus*.

**Additional file 6: Table S5.** Results of extended univariate LMMs for δ^13^C and δ^15^N of *Rattus rattus*.

**Additional file 7: Table S6.** Results of univariate LMMs for δ^13^C and δ^15^N of *Rattus rattus* sampled in Ambalafary, Antsahatsaka, Besakay, and Sahavarina.

**Additional file 8: Table S7.** Raw stable carbon and nitrogen data of soil samples.

**Additional file 9: Table S8.** Raw stable carbon and nitrogen data of plant samples.
