# Supplementary material for: Broad and flexible stable isotope niches in invasive non-native Rattus spp. in anthropogenic and natural habitats of central eastern Madagascar
Source: BMC Ecol. 2017 Apr 17;17:16. doi: 10.1186/s12898-017-0125-0 (PMC5393019; doi:10.1186/s12898-017-0125-0)
Supplement: Supplementary file 1 — Additional file 1: Text S1. Characterization of sampling sites. [file 12898_2017_125_MOESM1_ESM.doc]

**Additional files**

**Broad and flexible stable isotope niches in invasive non-native *Rattus* spp. in anthropogenic and natural habitats of central eastern Madagascar**

Melanie Dammhahn^1*^, Toky M. Randriamoria^2,3^, Steven M. Goodman^2,4^

^1^Animal Ecology, Institute for Biochemistry and Biology, Faculty of Natural Sciences, University of Potsdam, Maulbeerallee 1, 14469 Potsdam, Germany

^2^Association Vahatra, BP 3972, Antananarivo 101, Madagascar

^3^Département de Biologie Animale, Université d’Antananarivo, BP 906, Antananarivo 101, Madagascar

^4^Field Museum of Natural History, 1400 South Lake Shore Drive, Chicago, Illinois 60605, USA

^*^Corresponding author: melanie.dammhahn@uni-potsdam.de

**S1 Text: Characterization of sampling sites**

Non-primate terrestrial small mammal surveys were carried out across 12 sampling sites from July 2013 to March 2015 in the Moramanga District (Alaotra Mangoro Region) central eastern Madagascar (S1 Table). Sampling sites included natural forests and village settings or combinations of both.

**1. Natural forest sites**

**Antavibe**

This site is located about 1 km southeast of Mangiritsiry (village). This degraded forest is characterized by a semi-open canopy, generally 10-14 m in height, dense understory, and abundant lianas. Herbaceous cover is mainly discontinuous. The invasive shrubs *Clidemia hirta* (Family Melastomataceae) and *Lantana camara* (Family Verbenaceae) are common inside the forest, even 100 m into the interior. The Ranomena River flows through this forest.

**Avondrona**

This locality is partly situated about 4.5 km northwest of Antaniditra (village) and 7 km southeast of the Ambatovy mine site. This forest is slightly disturbed with trees 12-16 m in height and emergent trees up to 20 m. In general, the canopy is semi-open and the understory is dense. The site is also characterized by high abundance of *Pandanus* spp. (Family Pandanaceae) and lianas. Herbaceous cover is often dense, but discontinuous. Permanent streams occur in the forest.

**Lakato**

The site is about 1 km southeast of Ambalafary (village). This slightly degraded forest is characterized by a semi-open canopy rising to 10-15 m. Understory trees are common and herbaceous cover is dense and discontinuous. The Anivomaro River passes throughout the northern part of the site, which includes numerous temporary streams during the rainy season.

**Sahandambo**

The site is about 5.6 km northeast of Andasibe (village). This forest is slightly degraded with canopy trees between 10-15 m in height and emergent trees about 20 m. The canopy is generally semi-open and the understory dense. *Pandanus* and lianas are abundant.

**2. Sampling sites in village settings**

**Ambalafary**

This village is located about 1 km south of Ambodiriana (village). Ambalafary includes less than 20 closely packed houses surrounded by a range of habitats, including *Eucalyptus* plantation, *savoka* (regenerating areas of cleared forest), and *tavy* (hill rice); no irrigated paddy rice occurs in the immediate vicinity. The Sahantandra River passes by the village.

**Antanambao**

This village is about 2.5 km south of Ampasimpotsy (village) and is composed of about 20 dispersed houses and different local habitats, including irrigated paddy rice, *Eucalyptus* plantation, and agricultural fields.

**Antsahatsaka**

This village is along Route Nationale 2 and located about 3.4 km direct distance southeast of Moramanga and is made up of about 40 scattered houses. Sampled local habitats include irrigated paddy rice, *tavy*, agricultural crop fields, *savoka*, and *Eucalyptus* and bamboo plantations.

**Antsirinala**

This village is about 11 km northwest of Moramanga. The site is not heavily populated and contains about 10 widely dispersed houses. The immediate area is dominated by anthropogenic steppe and other sampled habitats include irrigated paddy rice, *savoka*, Ericaceae bush, agricultural crop fields, and *Eucalyptus* plantation.

**Maridaza**

The village of Maridaza is found about 4.5 km northeast of Ankarahara (village) and is composed of about 15 closely placed houses. The following habitats were sampled: savanna, agricultural crop fields, *savoka*, irrigated paddy rice, and *Eucalyptus, Mimosa,* and *Pinus* plantations.

**3. Combination of natural forest and village settings**

This category includes villages occurring in close proximity to natural forests (evergreen humid forest at mid elevation 800-1800 m; Du Puy & Moat, 1996). For these sites, sampling was conducted simultaneously in forested areas and secondary-anthropogenic vegetation in close vicinity to villages.

**Besakay**

The site is about 4.7 km northeast of Andasibe (village) and is composed of approximately 10 widely dispersed houses and about 100 m from natural forest, connected to the Parc National de Mantadia. The local forest is degraded and characterized by trees of up to 10-14 m in height, mainly open canopy, and relatively dense understory. Herbaceous cover is largely discontinuous. Different types of secondary habitats were sampled in the immediate vicinity of forested areas, including mainly agricultural fields (cassava and maize) and *savoka*.

**Mahatsara**

The site is located about 7 km north of Andasibe (village) and is composed of about 40 densely packed houses along the western limit of the Parc National de Mantadia. The sampled habitats included highly degraded forest, *savoka*, and irrigated paddy rice.

**Sahavarina**

The site is about 1.5 km north of Sahavarina (village) and includes about 30 scattered houses in an area above irrigated paddy rice. In addition, ericaceous bush and agricultural fields (banana and sugar cane) surround the rice paddy, which were also sampled, together with two adjacent degraded natural forest blocks.
