# Supplementary material for: Broad and flexible stable isotope niches in invasive non-native Rattus spp. in anthropogenic and natural habitats of central eastern Madagascar
Source: BMC Ecol. 2017 Apr 17;17:16. doi: 10.1186/s12898-017-0125-0 (PMC5393019; doi:10.1186/s12898-017-0125-0)
Supplement: Supplementary file 2 — Additional file 2: Table S1. Summary of the sampling period and geographic position for each sampling site. [file 12898_2017_125_MOESM2_ESM.doc]

**Additional files**

**Broad and flexible stable isotope niches in invasive non-native *Rattus* spp. in anthropogenic and natural habitats of central eastern Madagascar**

Melanie Dammhahn^1*^, Toky M. Randriamoria^2,3^, Steven M. Goodman^2,4^

^1^Animal Ecology, Institute for Biochemistry and Biology, Faculty of Natural Sciences, University of Potsdam, Maulbeerallee 1, 14469 Potsdam, Germany

^2^Association Vahatra, BP 3972, Antananarivo 101, Madagascar

^3^Département de Biologie Animale, Université d’Antananarivo, BP 906, Antananarivo 101, Madagascar

^4^Field Museum of Natural History, 1400 South Lake Shore Drive, Chicago, Illinois 60605, USA

^*^Corresponding author: melanie.dammhahn@uni-potsdam.de

**S1 Table:** Summary of the sampling period and geographic position for each sampling site. The period from July to October is defined as the dry season and from December to April as the rainy season. “Fokontany” is the basis of Malagasy administrative subdivision. “Commune rurale” is a rural administrative subdivision that includes several “Fokontany”.

| **TYPE OF SITE** | **SITE NAME** | **SAMPLING PERIOD** | **COMMUNE RURALE** | **FOKONTANY** | **LATITUDE** | **LONGITUDE** | **AVERAGE ALTITUDE**  **(m)** |
| --- | --- | --- | --- | --- | --- | --- | --- |
| **NATURAL FOREST** | Antavibe | 26-30 Sep 2014 | Ambohibary | Antsily | 19˚01'57.7'' | 48˚09'57.6'' | 900 |
|  | Avondrona | 21-25 Feb 2014 | Andasibe | Menalamba | 18˚50'25.5'' | 48˚20'46.7'' | 975 |
|  |  | 02-06 Aug 2014 |  |  |  |  |  |
|  | Lakato | 31 July-04 Aug 2013 | Ampasimpotsy Gara | Ambodiriana | 19˚02'38.0'' | 48˚20'55.0'' | 1010 |
|  |  | 21-25 Mar 2014 |  |  |  |  |  |
|  | Sahandambo | 14-18 Dec 2014 | Andasibe | Mahatsara | 19˚50'58.8'' | 48˚25'17.6'' | 1070 |
| **VILLAGE** | Ambalafary | 05-09 Oct 2013 | Ampasimpotsy Gara | Ambodiriana | 19˚02'00.4'' | 48˚20'20.3'' | 980 |
|  |  | 12-16 Apr 2014 |  |  |  |  |  |
|  | Antanambao | 25-29 Jan 2015 | Ampasimpotsy Gara | Ambodiriana | 18˚59'24.7' | 48˚20'49.2'' | 950 |
|  | Antsahatsaka | 24-28 Aug 2013 | Ampasimpotsy Gara | Tsiazompody | 18˚57'20.6'' | 48˚16'47.3'' | 940 |
|  |  | 25-29 Jan 2014 |  |  |  |  |  |
|  | Antsirinala | 1-17 Sep 2013 | Ambohibary | Antsirinala | 18˚53'29.1'' | 48˚09'10.0'' | 920 |
|  | Maridaza | 06-10 Sep 2014 | Ambohibary | Ankarahara | 18˚50'58.7'' | 48˚08'36.0'' | 910 |
|  |  | 07-11 Jan 2015 |  |  |  |  |  |
| **NATURAL FOREST-VILLAGE** | Besakay | 6-10 Jul 2013 | Andasibe | Andasifahatelo | 18˚54'04.2'' | 48˚27'00.0'' | 985 |
|  |  | 12-16 Jan 2014 |  |  |  |  |  |
|  |  | 21-25 Aug 2014 |  |  |  |  |  |
|  |  | 19-23 Mar 2015 | Andasibe | Andasifahatelo | 18˚54'04.2'' | 48˚27'00.0'' | 985 |
|  | Mahatsara | 01-05 Mar 2015 | Andasibe | Mahatsara | 18˚51'41.7'' | 48˚25'26.8'' | 950 |
|  | Sahavarina | 07-11 Mar 2014 | Andasibe | Menalamba | 18˚51'48.3'' | 48˚21'23.9'' | 925 |
|  |  | 17-21 Jul 2014 |  |  |  |  |  |
