# Supplementary material for: Broad and flexible stable isotope niches in invasive non-native Rattus spp. in anthropogenic and natural habitats of central eastern Madagascar
Source: BMC Ecol. 2017 Apr 17;17:16. doi: 10.1186/s12898-017-0125-0 (PMC5393019; doi:10.1186/s12898-017-0125-0)
Supplement: Supplementary file 3 — Additional file 3: Table S2. Areas of standard ellipses and convex hulls for Rattus rattus in different habitats. [file 12898_2017_125_MOESM3_ESM.doc]

**Additional files**

**Broad and flexible stable isotope niches in invasive non-native *Rattus* spp. in anthropogenic and natural habitats of central eastern Madagascar**

Melanie Dammhahn^1*^, Toky M. Randriamoria^2,3^, Steven M. Goodman^2,4^

^1^Animal Ecology, Institute for Biochemistry and Biology, Faculty of Natural Sciences, University of Potsdam, Maulbeerallee 1, 14469 Potsdam, Germany

^2^Association Vahatra, BP 3972, Antananarivo 101, Madagascar

^3^Département de Biologie Animale, Université d’Antananarivo, BP 906, Antananarivo 101, Madagascar

^4^Field Museum of Natural History, 1400 South Lake Shore Drive, Chicago, Illinois 60605, USA

^*^Corresponding author: melanie.dammhahn@uni-potsdam.de

**Within-species niche variation in *Rattus rattus* – additional results**

**S2 Table.** Areas of standard ellipses and convex hulls for *Rattus rattus* in different habitats. The stable isotope niche width of *R. rattus* was larger in natural forests as compared to agricultural fields and anthropogenic steppe. Shown are areas of standard ellipses and convex hulls in ‰² calculated with the R package *SIAR* (Parnell et al. 2010).

| **Variables** | **Natural forest** | **Agricultural fields** | **Anthropogenic steppe** |
| --- | --- | --- | --- |
| Area of the standard ellipse (SEA) | 14.98 | 11.82 | 14.15 |
| Area of convex hull (TA) | 79.91 | 51.36 | 77.91 |
