# Supplementary material for: Broad and flexible stable isotope niches in invasive non-native Rattus spp. in anthropogenic and natural habitats of central eastern Madagascar
Source: BMC Ecol. 2017 Apr 17;17:16. doi: 10.1186/s12898-017-0125-0 (PMC5393019; doi:10.1186/s12898-017-0125-0)
Supplement: Supplementary file 4 — Additional file 4: Table S3. Pairwise overlap between stable isotope niches of Rattus rattus in different habitats. [file 12898_2017_125_MOESM4_ESM.doc]

**Additional files**

**Broad and flexible stable isotope niches in invasive non-native *Rattus* spp. in anthropogenic and natural habitats of central eastern Madagascar**

Melanie Dammhahn^1*^, Toky M. Randriamoria^2,3^, Steven M. Goodman^2,4^

^1^Animal Ecology, Institute for Biochemistry and Biology, Faculty of Natural Sciences, University of Potsdam, Maulbeerallee 1, 14469 Potsdam, Germany

^2^Association Vahatra, BP 3972, Antananarivo 101, Madagascar

^3^Département de Biologie Animale, Université d’Antananarivo, BP 906, Antananarivo 101, Madagascar

^4^Field Museum of Natural History, 1400 South Lake Shore Drive, Chicago, Illinois 60605, USA

^*^Corresponding author: melanie.dammhahn@uni-potsdam.de

**S3 Table.** Pairwise overlap between stable isotope niches of *Rattus rattus* in different habitats. Shown are percent overlap in % of Bayesian standard ellipses, estimated with the R package *SIAR* (Parnell et al. 2010).

| **Overlap** | **Natural forests** | **Agricultural field** | **Anthropogenic steppe** |
| --- | --- | --- | --- |
| Natural forest |  | 56.5 | 72.1 |
| Agricultural field | 44.9 |  | 54.0 |
| Anthropogenic steppe | 68.0 | 64.1 |  |
