# Supplementary material for: Broad and flexible stable isotope niches in invasive non-native Rattus spp. in anthropogenic and natural habitats of central eastern Madagascar
Source: BMC Ecol. 2017 Apr 17;17:16. doi: 10.1186/s12898-017-0125-0 (PMC5393019; doi:10.1186/s12898-017-0125-0)
Supplement: Supplementary file 5 — Additional file 5: Table S4. Results of extended multivariate Bayesian mixed model for δ13C and δ15N of Rattus rattus. [file 12898_2017_125_MOESM5_ESM.doc]

**Additional files**

**Broad and flexible stable isotope niches in invasive non-native *Rattus* spp. in anthropogenic and natural habitats of central eastern Madagascar**

Melanie Dammhahn^1*^, Toky M. Randriamoria^2,3^, Steven M. Goodman^2,4^

^1^Animal Ecology, Institute for Biochemistry and Biology, Faculty of Natural Sciences, University of Potsdam, Maulbeerallee 1, 14469 Potsdam, Germany

^2^Association Vahatra, BP 3972, Antananarivo 101, Madagascar

^3^Département de Biologie Animale, Université d’Antananarivo, BP 906, Antananarivo 101, Madagascar

^4^Field Museum of Natural History, 1400 South Lake Shore Drive, Chicago, Illinois 60605, USA

^*^Corresponding author: melanie.dammhahn@uni-potsdam.de

**S4 Table.** Results of extended multivariate Bayesian mixed model for δ^13^C and δ^15^N of *Rattus rattus*. Shown are posterior means, lower and upper 95% credibility intervals, and P-values, which are based on 10,000 simulations.

| **Parameter** | **Posterior mean** | **l-95% CI** | **u-95% CI** | **p** |
| --- | --- | --- | --- | --- |
| Intercept δ^13^C | **-22.55** | **-23.15** | **-21.80** | **<0.001** |
| Intercept δ^15^N | **7.19** | **5.88** | **8.31** | **<0.001** |
| Anthropogenic steppe^1^ | 0.16 | -0.15 | 0.47 | 0.306 |
| **Agricultural field^1^** | **0.43** | **0.04** | **0.82** | **0.032** |
| Sex² | -0.15 | -0.36 | 0.07 | 0.172 |
| Age class^3^ | -0.01 | -0.22 | 0.22 | 0.918 |
| Season^4^ | -0.23 | -0.49 | 0.04 | 0.088 |

Reference levels are ^1^natural forest, ²female, ^3^parous, and ^4^dry season.
