# Supplementary material for: Broad and flexible stable isotope niches in invasive non-native Rattus spp. in anthropogenic and natural habitats of central eastern Madagascar
Source: BMC Ecol. 2017 Apr 17;17:16. doi: 10.1186/s12898-017-0125-0 (PMC5393019; doi:10.1186/s12898-017-0125-0)
Supplement: Supplementary file 6 — Additional file 6: Table S5. Results of extended univariate LMMs for δ13C and δ15N of Rattus rattus. [file 12898_2017_125_MOESM6_ESM.doc]

**Additional files**

**Broad and flexible stable isotope niches in invasive non-native *Rattus* spp. in anthropogenic and natural habitats of central eastern Madagascar**

Melanie Dammhahn^1*^, Toky M. Randriamoria^2,3^, Steven M. Goodman^2,4^

^1^Animal Ecology, Institute for Biochemistry and Biology, Faculty of Natural Sciences, University of Potsdam, Maulbeerallee 1, 14469 Potsdam, Germany

^2^Association Vahatra, BP 3972, Antananarivo 101, Madagascar

^3^Département de Biologie Animale, Université d’Antananarivo, BP 906, Antananarivo 101, Madagascar

^4^Field Museum of Natural History, 1400 South Lake Shore Drive, Chicago, Illinois 60605, USA

^*^Corresponding author: melanie.dammhahn@uni-potsdam.de

**S5 Table.** Results of extended univariate LMMs for δ^13^C and δ^15^N of *Rattus rattus*. Significant results are marked in bold.

| **Parameter** | **β ± SE** | ***t*** | ***P*** | ***Χ²**** | ***P**** |
| --- | --- | --- | --- | --- | --- |
| δ^13^C |  |  |  |  |  |
| Intercept | **-22.87 ± 0.30** | **76.55** | **<0.001** |  |  |
| Anthropogenic steppe^1^ | **0.62 ± 0.25** | **2.54** | **0.011** |  |  |
| Agricultural field^1^ | **0.67 ± 0.32** | **2.11** | **0.035** | **6.99**^5^ | **0.030**^5^ |
| Sex² | -0.10 ± 0.19 | 0.51 | 0.610 | 0.25 | 0.616 |
| Age class^3^ | 0.16 ± 0.20 | 0.81 | 0.417 | 0.80 | 0.371 |
| Season^4^ | -0.28 ± 0.22 | 1.28 | 0.202 | 2.67 | 0.103 |
|  |  |  |  |  |  |
| δ^15^N |  |  |  |  |  |
| Intercept | **7.31 ± 0.56** | **13.10** | **<0.001** |  |  |
| Anthropogenic steppe^1^ | 0.01 ± 0.18 | 1.51 | 0.973 |  |  |
| Agricultural field^1^ | 0.34 ± 0.26 | 1.34 | 0.129 | 3.39^5^ | 0.184^5^ |
| Sex² | -0.17 ± 0.12 | 1.34 | 0.181 | 2.18 | 0.139 |
| Age class^3^ | -0.09 ± 0.13 | 0.70 | 0.484 | 0.50 | 0.478 |
| Season^4^ | -0.22 ± 0.16 | 1.43 | 0.153 | 1.08 | 0.298 |

Reference levels are ^1^natural forest, ²female, ^3^parous, and ^4^dry season.

**Χ²* and *P*-values are based on Log-likelihood-ratio tests (LRT) comparing nested models with and without the respective term with *df*= 1; ^5^for the main effect of habitat type with *df* = 2.
