# Supplementary material for: Broad and flexible stable isotope niches in invasive non-native Rattus spp. in anthropogenic and natural habitats of central eastern Madagascar
Source: BMC Ecol. 2017 Apr 17;17:16. doi: 10.1186/s12898-017-0125-0 (PMC5393019; doi:10.1186/s12898-017-0125-0)
Supplement: Supplementary file 7 — Additional file 7: Table S6. Results of univariate LMMs for δ13C and δ15N of Rattus rattus sampled in Ambalafary, Antsahatsaka, Besakay, and Sahavarina. [file 12898_2017_125_MOESM7_ESM.doc]

**Additional files**

**Broad and flexible stable isotope niches in invasive non-native *Rattus* spp. in anthropogenic and natural habitats of central eastern Madagascar**

Melanie Dammhahn^1*^, Toky M. Randriamoria^2,3^, Steven M. Goodman^2,4^

^1^Animal Ecology, Institute for Biochemistry and Biology, Faculty of Natural Sciences, University of Potsdam, Maulbeerallee 1, 14469 Potsdam, Germany

^2^Association Vahatra, BP 3972, Antananarivo 101, Madagascar

^3^Département de Biologie Animale, Université d’Antananarivo, BP 906, Antananarivo 101, Madagascar

^4^Field Museum of Natural History, 1400 South Lake Shore Drive, Chicago, Illinois 60605, USA

^*^Corresponding author: melanie.dammhahn@uni-potsdam.de

**Habitat mismatching – additional results**

**S6 Table.** Results of univariate LMMs for δ^13^C and δ^15^N of *Rattus rattus* sampled at Ambalafary, Antsahatsaka, Besakay, and Sahavarina, i.e. including only sites with > 5 samples per habitat type (*N* = 4 sites, *N* = 345 individuals). Significant results are marked in bold.

| **Parameter** | **β ± SE** | ***t*** | ***P*** | ***Χ²**** | ***P**** |
| --- | --- | --- | --- | --- | --- |
| δ^13^C |  |  |  |  |  |
| Intercept | **-22.93 ± 0.33** | **67.86** | **<0.001** |  |  |
| Anthropogenic steppe^1^ | **0.55 ± 0.29** | **1.91** | **0.055** |  |  |
| Agricultural field^1^ | **0.69 ± 0.34** | **2.00** | **0.045** | **5.32**^5^ | **0.070**^5^ |
|  |  |  |  |  |  |
| δ^15^N |  |  |  |  |  |
| Intercept | **6.69 ± 0.74** | **9.10** | **<0.001** |  |  |
| Anthropogenic steppe^1^ | 0.21 ± 0.20 | 1.00 | 0.302 |  |  |
| Agricultural field^1^ | 0.56 ± 0.24 | 2.29 | 0.022 | 5.30^5^ | 0.071^5^ |

Reference level is ^1^natural forest. **Χ²* and *P*-values are based on Log-likelihood-ratio tests (LRT) comparing nested models with and without the main effect of habitat type with *df*= 2.
