# Supplementary material for: Broad and flexible stable isotope niches in invasive non-native Rattus spp. in anthropogenic and natural habitats of central eastern Madagascar
Source: BMC Ecol. 2017 Apr 17;17:16. doi: 10.1186/s12898-017-0125-0 (PMC5393019; doi:10.1186/s12898-017-0125-0)
Supplement: Supplementary file 8 — Additional file 8: Table S7. Raw stable carbon and nitrogen data of soil samples. [file 12898_2017_125_MOESM8_ESM.doc]

**Additional files**

**Broad and flexible stable isotope niches in invasive non-native *Rattus* spp. in anthropogenic and natural habitats of central eastern Madagascar**

Melanie Dammhahn^1*^, Toky M. Randriamoria^2,3^, Steven M. Goodman^2,4^

^1^Animal Ecology, Institute for Biochemistry and Biology, Faculty of Natural Sciences, University of Potsdam, Maulbeerallee 1, 14469 Potsdam, Germany

^2^Association Vahatra, BP 3972, Antananarivo 101, Madagascar

^3^Département de Biologie Animale, Université d’Antananarivo, BP 906, Antananarivo 101, Madagascar

^4^Field Museum of Natural History, 1400 South Lake Shore Drive, Chicago, Illinois 60605, USA

^*^Corresponding author: melanie.dammhahn@uni-potsdam.de

**Stable carbon and nitrogen signatures of soil and plant samples**

**Table S7. Raw stable carbon and nitrogen data of soil samples** in ‰ collected at the different sampling sites (see description above) and habitat types

| **Sampling site** | **Habitat type** | **δ^13^C [‰]** | **δ^15^N [‰]** |
| --- | --- | --- | --- |
| Ambalafary | Natural forest | -28.5 | -0.5 |
| Ambalafary | Natural forest | -26.7 | 4.0 |
| Ambalafary | Natural forest | -25.7 | 2.9 |
| Ambalafary | Anthropogenic steppe | -25.3 | 3.2 |
| Ambalafary | Anthropogenic steppe | -25.6 | 6.3 |
| Ambalafary | Natural forest | -24.9 | 5.9 |
| Antavibe | Natural forest | -26.0 | 7.5 |
| Antavibe | Natural forest | -25.4 | 7.9 |
| Antavibe | Natural forest | -26.5 | 7.5 |
| Antavibe | Natural forest | -26.6 | 6.4 |
| Antavibe | Natural forest | -26.2 | 6.9 |
| Antavibe | Natural forest | -26.7 | 7.0 |
| Antsahatsaka | Agricultural field | -25.6 | 7.6 |
| Antsahatsaka | Agricultural field | -25.6 | 8.1 |
| Antsahatsaka | Natural forest | -24.7 | 8.3 |
| Antsahatsaka | Natural forest | -24.5 | 6.4 |
| Antsahatsaka | Natural forest | -22.8 | 9.0 |
| Antsahatsaka | Natural forest | -24.7 | 6.4 |
| Antsirinala | Anthropogenic steppe | -21.5 | 9.1 |
| Antsirinala | Anthropogenic steppe | -21.4 | 6.4 |
| Antsirinala | Natural forest | -21.7 | 7.8 |
| Avondrona | Natural forest | -26.4 | 7.7 |
| Avondrona | Natural forest | -25.9 | 8.8 |
| Avondrona | Natural forest | -26.6 | 8.3 |
| Besakay | Anthropogenic steppe | -26.2 | 5.2 |
| Besakay | Anthropogenic steppe | -26.0 | 4.4 |
| Besakay | Anthropogenic steppe | -26.0 | 3.8 |
| Besakay | Agricultural field | -26.3 | 3.9 |
| Besakay | Agricultural field | -26.1 | 5.7 |
| Besakay | Agricultural field | -25.9 | 5.1 |
| Besakay | Natural forest | -27.1 | 2.2 |
| Besakay | Natural forest | -27.1 | 3.3 |
| Besakay | Natural forest | -27.0 | 2.2 |
| Mahatsara | Anthropogenic steppe | -24.7 | 7.0 |
| Mahatsara | Agricultural field | -23.3 | 6.0 |
| Mahatsara | Natural forest | -25.5 | 6.9 |
| Maridaza | Anthropogenic steppe | -21.5 | 8.4 |
| Maridaza | Anthropogenic steppe | -24.7 | 6.6 |
| Maridaza | Agricultural field | -17.8 | 7.7 |
| Sahandambo | Natural forest | -27.3 | 3.6 |
| Sahandambo | Natural forest | -26.4 | 7.1 |
| Sahandambo | Natural forest | -26.9 | 3.8 |
| Sahavarina | Anthropogenic steppe | -18.8 | 8.9 |
| Sahavarina | Agricultural field | -20.1 | 7.4 |
| Sahavarina | Natural forest | -26.5 | 5.2 |
| Sahavarina | Natural forest | -24.7 | 6.0 |
| Tabakol | Agricultural field | -24.8 | 8.4 |
| Tabakol | Agricultural field | -27.0 | 4.9 |
| Tabakol | Natural forest | -25.4 | 6.5 |
