# Supplementary material for: Broad and flexible stable isotope niches in invasive non-native Rattus spp. in anthropogenic and natural habitats of central eastern Madagascar
Source: BMC Ecol. 2017 Apr 17;17:16. doi: 10.1186/s12898-017-0125-0 (PMC5393019; doi:10.1186/s12898-017-0125-0)
Supplement: Supplementary file 9 — Additional file 9: Table S8. Raw stable carbon and nitrogen data of plant samples. [file 12898_2017_125_MOESM9_ESM.doc]

**Additional files**

**Broad and flexible stable isotope niches in invasive non-native *Rattus* spp. in anthropogenic and natural habitats of central eastern Madagascar**

Melanie Dammhahn^1*^, Toky M. Randriamoria^2,3^, Steven M. Goodman^2,4^

^1^Animal Ecology, Institute for Biochemistry and Biology, Faculty of Natural Sciences, University of Potsdam, Maulbeerallee 1, 14469 Potsdam, Germany

^2^Association Vahatra, BP 3972, Antananarivo 101, Madagascar

^3^Département de Biologie Animale, Université d’Antananarivo, BP 906, Antananarivo 101, Madagascar

^4^Field Museum of Natural History, 1400 South Lake Shore Drive, Chicago, Illinois 60605, USA

^*^Corresponding author: melanie.dammhahn@uni-potsdam.de

**Table S8. Raw stable carbon and nitrogen data of plant samples** in ‰ collected at the different sampling sites (see description above) and habitat types

| **Sampling site** | **Habitat type** | **δ^13^C [‰]** | **δ^15^N [‰]** |
| --- | --- | --- | --- |
| Ambalafary | Natural forest | -30.8 | -0.5 |
| Ambalafary | Natural forest | -29.9 | -1.7 |
| Ambalafary | Natural forest | -34.3 | 0.6 |
| Ambalafary | Natural forest | -30.8 | 2.1 |
| Ambalafary | Anthropogenic steppe | -29.8 | 2.3 |
| Ambalafary | Anthropogenic steppe | -28.4 | 5.2 |
| Ambalafary | Anthropogenic steppe | -12.8 | 1.6 |
| Ambalafary | Anthropogenic steppe | -27.8 | 3.9 |
| Ambalafary | Anthropogenic steppe | -28.8 | 2.6 |
| Ambalafary | Anthropogenic steppe | -29.2 | 3.6 |
| Ambalafary | Anthropogenic steppe | -28.6 | 0.6 |
| Ambalafary | Natural forest | -30.7 | 1.0 |
| Antavibe | Natural forest | -31.0 | -0.4 |
| Antavibe | Natural forest | -29.1 | 3.2 |
| Antavibe | Natural forest | -30.6 | 4.6 |
| Antavibe | Natural forest | -30.6 | 1.5 |
| Antavibe | Natural forest | -30.2 | 3.7 |
| Antavibe | Natural forest | -28.2 | 1.2 |
| Antsahatsaka | Anthropogenic steppe | -29.0 | 2.5 |
| Antsahatsaka | Natural forest | -28.7 | 2.9 |
| Antsirinala | Anthropogenic steppe | -29.7 | 0.5 |
| Avondrona | Natural forest | -31.2 | 4.9 |
| Avondrona | Natural forest | -27.7 | 2.0 |
| Avondrona | Natural forest | -30.4 | 4.7 |
| Avondrona | Natural forest | -30.2 | 1.3 |
| Avondrona | Natural forest | -30.9 | 3.3 |
| Avondrona | Natural forest | -30.2 | 1.4 |
| Besakay | Anthropogenic steppe | -27.6 | 1.6 |
| Besakay | Anthropogenic steppe | -26.7 | 0.5 |
| Besakay | Anthropogenic steppe | -27.9 | 0.6 |
| Besakay | Anthropogenic steppe | -28.8 | -1.9 |
| Besakay | Anthropogenic steppe | -29.9 | -2.2 |
| Besakay | Natural forest | -26.0 | 4.7 |
| Besakay | Natural forest | -27.8 | -2.3 |
| Besakay | Natural forest | -29.3 | -2.5 |
| Besakay | Natural forest | -29.6 | 0.3 |
| Mahatsara | Agricultural field | -30.8 | 1.0 |
| Mahatsara | Anthropogenic steppe | -31.5 | 0.5 |
| Mahatsara | Natural forest | -26.5 | -4.2 |
| Maridaza | Anthropogenic steppe | -29.1 | 3.2 |
| Maridaza | Anthropogenic steppe | -31.0 | 4.4 |
| Maridaza | Natural forest | -29.5 | -0.5 |
| Maridaza | Natural forest | -30.8 | 2.7 |
| Sahandambo | Natural forest | -31.2 | 0.3 |
| Sahandambo | Natural forest | -30.3 | -0.2 |
| Sahandambo | Natural forest | -28.9 | 1.2 |
| Sahavarina | Agricultural field | -27.6 | 4.6 |
| Sahavarina | Agricultural field | -29.6 | 9.6 |
| Sahavarina | Agricultural field | -12.6 | 2.2 |
| Sahavarina | Anthropogenic steppe | -30.3 | -0.8 |
| Sahavarina | Anthropogenic steppe | -28.6 | -0.4 |
| Sahavarina | Natural forest | -31.0 | 0.1 |
| Sahavarina | Natural forest | -28.9 | 3.7 |
| Sahavarina | Natural forest | -28.7 | -1.4 |
| Tabakol | Agricultural field | -13.4 | 5.1 |
| Tabakol | Anthropogenic steppe | -29.0 | 5.9 |
| Tabakol | Natural forest | -28.5 | 3.5 |
